# Molecular mechanisms for mediating light-dependent nucleo/cytoplasmic partitioning of phytochrome photoreceptors

**DOI:** 10.1111/nph.13207

**Published:** 2014-12-15

**Authors:** Cornelia Klose, András Viczián, Stefan Kircher, Eberhard Schäfer, Ferenc Nagy

**Affiliations:** 1 Institute of Botany, University of FreiburgSchänzlestrasse 1, D-79104, Freiburg, Germany; 2 Institute of Plant Biology, Biological Research CentreTemesvári krt. 62, H-6726, Szeged, Hungary; 3 School of Biological Sciences, Institute of Molecular Plant Science, University of EdinburghEdinburgh, EH9 3JH, UK

**Keywords:** light signaling, nuclear body, nuclear import, nuclear translocation, photomorphogenesis, phytochrome

## Abstract

The photoreceptors phytochromes monitor the red/far-red part of the spectrum, exist in the biologically active Pfr (far-red absorbing) or inactive Pr (red absorbing) forms, and function as red/far-red light-regulated molecular switches to modulate plant development and growth. Phytochromes are synthesized in the cytoplasm, and light induces translocation of the Pfr conformer into the nucleus. Nuclear import of phytochromes is a highly regulated process and is fine-tuned by the quality and quantity of light. It appears that phytochrome A (phyA) and phytochrome B (phyB) do not possess active endogenous nuclear import signals (NLSs), thus light-induced translocation of these photoreceptors into the nucleus requires direct protein–protein interactions with their NLS-containing signaling partners. Sub-cellular partitioning of the various phytochrome species is mediated by different molecular machineries. Translocation of phyA into the nucleus is promoted by FAR-RED ELONGATED HYPOCOTYL 1 (FHY1) and FHY1-LIKE (FHL), but the identity of nuclear transport facilitators mediating the import of phyB-E into the nucleus remains elusive. Phytochromes localized in the nucleus are associated with specific protein complexes, termed photobodies. The size and distribution of these structures are regulated by the intensity and duration of irradiation, and circumstantial evidence indicates that they are involved in fine-tuning phytochrome signaling.

## Introduction

Plants are sessile organisms, and to ensure optimal growth they must adapt to changes in their environment. Light is one of the most variable abiotic environmental factors, and plants use light not only as the energy source for photosynthesis but also as an essential developmental cue. To monitor changes in the spectral composition, intensity, direction and duration of the sunlight, plants have evolved a battery of photoreceptors. These photoreceptors, including the red/far-red light absorbing phytochromes regulate various aspects of light-dependent development (photomorphogenesis) of plants from germination to seed setting. Phytochromes exist as dimers, and each monomer contains a covalently linked open tetra-pyrrole chain as chromophore (Rockwell *et al*., 2006). These photoreceptors are synthesized in their biologically inactive conformation (Pr, R-light absorbing form) in the cytosol and converted by light absorbance to the biologically active conformation (Pfr, FR-light absorbing form). Subsequent illumination by FR-light rapidly converts the Pfr back into Pr, but the Pfr conformer can also relax back into Pr by a slower thermal dynamic process called dark reversion. The absorption spectra of the Pfr and Pr conformers partially overlap thus phytochromes continuously cycle between Pfr and Pr. Due to this process a photoequilibrium established (Pfr : Ptot), thus the actual amount of Pfr is dependent on the spectral composition of the light environment (Rockwell *et al*., 2006). Based on their photobiological properties Arabidopsis phytochromes (phyA–phyE) can be divided into two groups: phyA is a highly specialized sensor that mediates the very low fluence response (VLFR) initiated by extreme low amounts of light and the high irradiation response (HIR) to continuous FR-light (Nagy & Schafer, 2002); phyA is the dominant phytochrome in etiolated seedlings and plays an essential role in regulating transition from skotomorphogenesis (development in the absence of light) to photomorphogenesis (development in the presence of light). In contrast to the light-labile phyA Pfr, the Pfr conformer of phyB, phyC, phyD and phyE is light-stable, and the action of phyB–phyE is inhibited by FR-light. Accordingly, phyB-phyE mediates, in a reversible fashion, the so called low fluence response (LFR) which is triggered by R light and terminated by FR-light (Nagy & Schafer, 2002).

At the molecular level, phytochrome-controlled photomorphogenesis is mediated by a complex signaling network. Recent studies established that it is the phyA–phyE Pfr localized in the nucleus that controls the overwhelming majority of developmental and growth responses and that translocation of phyA–phyE from the cytoplasm into the nucleus is an early and rate limiting step of R/FR induced signaling (for review see Fankhauser & Chen, 2008). Thus it is generally accepted that light-induced redistribution of phyA-phyE is required to inactivate the CONSTITUTIVELY PHOTOMORPHOGENIC 1 (COP1)/SUPPRESSOR OF PHYA-105 (SPA1) negative regulatory complex and to initiate degradation/inactivation of the basic helix-loop-helix (bHLH) type PHYTOCHROME INTERACTING FACTORS (PIFs) that themselves are negative regulators of light-induced signaling. Translocation of phytochromes into the nucleus is a light quality- and quantity-dependent process, but the molecular mechanism(s)/machinery(ies) mediating this highly divergent yet essential signaling step is not fully understood. The latest comprehensive review on this subject was published some years ago (Fankhauser & Chen, 2008). Thus, here we attempt to highlight those novel observations that have advanced our understanding about this cellular event critical for the action of these photoreceptors.

## Molecular mechanisms of phyA nuclear import

Nuclear accumulation of phyA is an indispensable step in phyA signaling and depends directly on the two plant-specific proteins, FHY1 (FAR-RED ELONGATED HYPOCOTYL 1) and FHL (FHY1-LIKE). FHY1 and FHL each contain a functional NLS (nuclear localization signal), a NES (nuclear export signal) and a phyA binding site in their N-terminal half. This short domain is both necessary and sufficient for the full functionality of these molecules (Genoud *et al*., 2008). FHY1 and FHL co-localize with phyA in the nucleus and in photobodies and interact with phyA preferentially in its light-activated Pfr form *in vitro* and *in vivo*. By contrast, co-immunoprecipitation assays showed that both FHY1 and FHL interact more stably with the Pr form of phyA in *Arabidopsis* seedlings (Shen *et al*., 2009). This latter finding is contradictory to many observations regarding the intracellular distribution of phyA and its physiological consequences. In fact, mathematical modeling suggests that association and dissociation of phyA to FHY1 and FHL are highly dynamic processes, thus it is conceivable that protein complexes purified by co-immunoprecipitation from whole seedlings do not automatically reflect the active signaling complexes *in vivo*. According to the currently accepted model, FHY1/FHL work as shuttle proteins for phyA nuclear import. They bind to phyA Pfr in the cytosol, transport it into the nucleus and recycle back into the cytosol as soon as phyA is converted to Pr (Genoud *et al*., 2008; Rausenberger *et al*., 2011). Two coupled photoconversion cycles of phyA, one in the cytosol and one in the nucleus, are required for optimal shuttling mechanism, and this works most effectively in far-red light (Rausenberger *et al*., 2011). Because the amount of phyA exceeds by far the amount of FHY1 and FHL in etiolated plants, nucleo-cytoplasmic shuttling of FHY1 and FHL is essential for accumulation of phyA in the nucleus. Mutations that interfere with FHY1/FHL binding or dissociation from phyA would disrupt recycling of FHY1/FHL and nuclear transport of phyA, thus are expected to reduce sensitivity to FR-light. Consistent with this assumption, the constitutively active phyA^Y242H^ exhibits a moderate *cop1* phenotype (partial de-etiolation) in darkness and a strong dominant-negative phenotype in FR-light, because phyA^Y242H^ binds to FHY1 permanently and blocks further nuclear phyA import. phyA^Y242H^ activity still requires FHY1 and FHL, because *fhy1 fhl* mutant seedlings expressing phyA^Y242H^ remain fully etiolated, whereas fusing an NLS directly to phyA^Y242H^ results in a strong *cop1* phenotype (nearly full de-etiolation) (Rausenberger *et al*., 2011).

Based on the above-described model, the amount of shuttling FHY1/FHL molecules plays a critical role in efficient phyA signaling. The expression level of *FHY1* and *FHL* depends on *FHY3* (*FAR-RED ELONGATED HYPOCOTYL 3*) and *FAR1* (*FAR-RED IMPAIRED RESPONSE 1*), as these TFs bind to the promoters of *FHY1* and *FHL* to upregulate their expression (Lin *et al*., 2007). The nuclear accumulation of phyA is abolished in the *fhy3 far1* double mutant but can be rescued by constitutive overexpression of *FHY1,* demonstrating that FHY3 and FAR1 indirectly control phyA nuclear transport (Lin *et al*., 2007), whereas phyA signaling by negative feedback regulation reduces the expression of *FHY1* and *FHL*. Molecular analysis of this feedback regulation demonstrated that phyA promotes the accumulation of the bZIP transcription factor HY5 (ELONGATED HYPOCOTYL 5) which binds to *FHY1/FHL* promoters, and via interacting with the positive regulators FHY3 and FAR1 negatively regulates *FHY1/FHL* gene expression (Li *et al*., 2010). Phosphorylation of FHY1 is another mechanism to modify phyA signaling. PhyA phosphorylates FHY1 (but not FHL) *in vitro* and the rapid R**-**dependent phosphorylation of FHY1 *in vivo* is not detectable in *phyA* null background (Shen *et al*., 2009; Chen *et al*., 2012). Phosphorylated FHY1 is the preferred substrate for proteasomal degradation, and although its binding to phyA is not abolished, it inhibits nuclear localization of the photoreceptor. Phosphorylation of FHY1 occurs preferentially when high Pfr concentrations are formed, thus it leads to inhibition of phyA signaling in red light and which contributes to the shift in phyA action towards far-red light (Chen *et al*., 2012).

Recently it was reported that PIF3 and PIF1 could mediate nuclear translocation of a phyA-N-terminal fragment in a cell-free import system (in a conformation-dependent fashion) (Pfeiffer *et al*., 2012). This finding indicates that a FHY1/FHL-independent phyA nuclear transport mechanism might operate *in planta,* although phyA was not detectable in nuclei of Arabidopsis seedlings lacking FHY1 and FHL. Consistent with the hypothesis of alternative nuclear import machinery, a few phyA-dependent nuclear responses were observed in *fhy1 fhl* but not in *phyA* mutants (Kami *et al*., 2012; Pfeiffer *et al*., 2012). In this context we note that phyA Pfr was shown to degrade in the cytoplasm and nuclei but the rate of degradation was higher in the nuclei (Debrieux & Fankhauser, 2010). Circumstantial evidence indicates that retention of phyA in the cytoplasm or export of phyA into the cytoplasm does not play significant role in regulating cellular distribution and abundance of the photoreceptor (Toledo-Ortiz *et al*., 2010).

**Figure 1:**
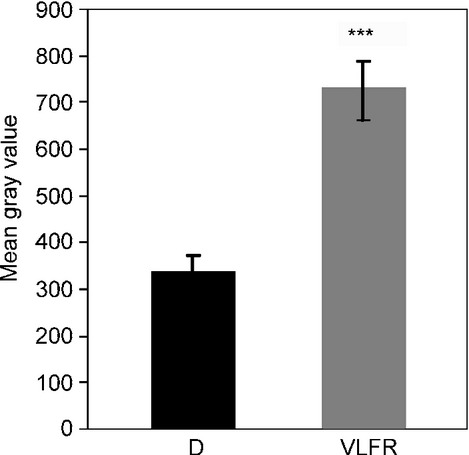
Quantification of phyA-YFP nuclear accumulation after a single very low fluence response (VLFR) pulse. Mean gray values of at least 20 nuclei were measured using epifluorescence microscopy images of 4-d-old etiolated *Arabidopsis thaliana* seedlings expressing *35S:PHYA-YFP* transgene in *phyA-211* mutant genetic background in darkness (D) or after a VLFR pulse (665 nm, 3 μmol m^−2^) (VLFR). Error bars, ± SE; ***, *P *< 0.001, compared with dark sample.

Phytochromes are dimeric proteins, thus depending on the light conditions they can exist as inactive Pr-Pr homodimers, and active Pfr-Pr hetero- and Pfr-Pfr homodimers. Fig.1 shows that phyA nuclear import is detectable after a VLFR pulse creating *c*. 1.5% Pfr. Under VLFR conditions the Pfr-Pfr homodimer pool is insignificant, thus it is the Pfr-Pr heterodimer which is imported into the nucleus and initiates signaling. In the current model for phyA nuclear photoconversion of one phyA monomer to Pfr would be sufficient to activate nuclear transport. Light conditions creating a high proportion of Pfr-Pfr homodimers induce the formation of sequestered areas of phytochrome (SAPs) in the cytosol in which Pfr-Pfr could be trapped. We hypothesize that SAP formation competes with phyA-Pfr binding to FHY1 or FHL and thereby interferes with nuclear import in red light, which then contributes to the far-red shift of the phyA action.

Phylogenetic analyses revealed that, similarly to phyA, the FHY1-like proteins exhibit a high degree of sequence conservation in angiosperms (Genoud *et al*., 2008). Cryptogam phytochromes evolved in parallel to seed plant phytochromes, but HIR-like responses to far-red light are not restricted to seed plants (Possart & Hiltbrunner, 2013). FHY1-like proteins are also present in cryptogams (ferns, mosses and green algae) and several cryptogam FHY1-like proteins are able to bind *Arabidopsis* phyA in a Pfr-specific way. Furthermore, Pp (*Physcomitrella patens*)-FHY1 was shown to be essential for nuclear import of Pp-PHY1 and is even functional in *Arabidopsis* (Possart & Hiltbrunner, 2013). It is conceivable that HIR-like responses had evolved before the divergence of seed plants and cryptogams and that HIR signaling, including FHY1-dependent nuclear import, is an ancient mechanism. This points towards a common molecular mechanism of phyA nuclear import in angiosperms and underlines the importance of regulated subcellular localization for phyA signaling.

## Molecular mechanisms of phyB nuclear import

phyB is the dominant R/FR receptor of light-grown plants, and the first pioneering studies addressing light-regulated translocation of any phytochromes into the nucleus were performed by using transgenic plants expressing phyB-GUS (β-GLUCURONIDASE) (Sakamoto & Nagatani, 1996) or phyB-GFP (GREEN FLUORESCENCE PROTEIN) fusion proteins (Kircher *et al*., 1999; Yamaguchi *et al*., 1999). Subsequent studies revealed the wavelength and fluence dependence of phyB nuclear import, and showed that this cellular event is essential for phyB signaling (Huq *et al*., 2003) and that phyB mutants defective in signaling do not form nuclear bodies (Kircher *et al*., 2002). These findings initiated further studies to identify the molecular machinery mediating nuclear import of phyB. It was concluded that: (1) PHYB contains NLS-like motif(s), but no typical NLS sequence(s) (Sakamoto & Nagatani, 1996); (2) these motifs are localized in the C-terminal domain, (Matsushita *et al*., 2003; Chen *et al*., 2005); (3) the C-terminal domain interacts with the N-terminal domain preferentially in the Pr form, and this interaction (4) severely reduces accessibility of the NLS-like motif of phyB Pr to interact with the importin-based nuclear import machinery. Accordingly, the generally accepted model explained the R/FR reversibility of phyB nuclear import by masking/unmasking the NLS-like motif(s) in the C-terminus via the Pr to Pfr conformation change.

Despite the fact that the postulated NLS-like motif(s) of phyB was never validated experimentally, the view about the nuclear import of phyB did not change until the report published by Pfeiffer *et al*. (2012). These authors showed in the cell-free *Acetabularia* system that (1) phyB was excluded from the nucleus, (2) PIF3 promoted nuclear import of phyB Pfr but not of phyB Pr and (3) a chimeric protein containing only the phyB binding site of PIF3 fused to an NLS motif was equally sufficient to facilitate selective translocation of phyB Pfr into the nucleus. Importantly, the same authors demonstrated that (1) nuclear import of phyB was impaired in the Arabidopsis quadruple PIF mutant (*pifq)* seedlings grown under low intensity of white light, whereas (2) nuclear accumulation of phyB in the same *pifq* mutant was not affected in saturating light. These observations indicated that biological function of PIFs in mediating nuclear import of phyB is limited (Pfeiffer *et al*., 2012). This conclusion is also supported by the data showing that (1) the constitutively active phyB^Y276H^ mutant in the absence of detectable amount of PIFs accumulates to high levels in the nucleus (Galvao *et al*., 2012) and (3) mutation abolishing binding of phyB to PIFs *in vitro* did not significantly alter nuclear localisation of the photoreceptor in transgenic plants (Oka *et al*., 2008). Independent of the limited function of PIFs the data reported by Pfeiffer *et al*. (2012)*,* suggest that translocation of phyB into the nucleus can also be mediated by NLS-bearing proteins interacting specifically with phyB Pfr.

**Figure 2:**
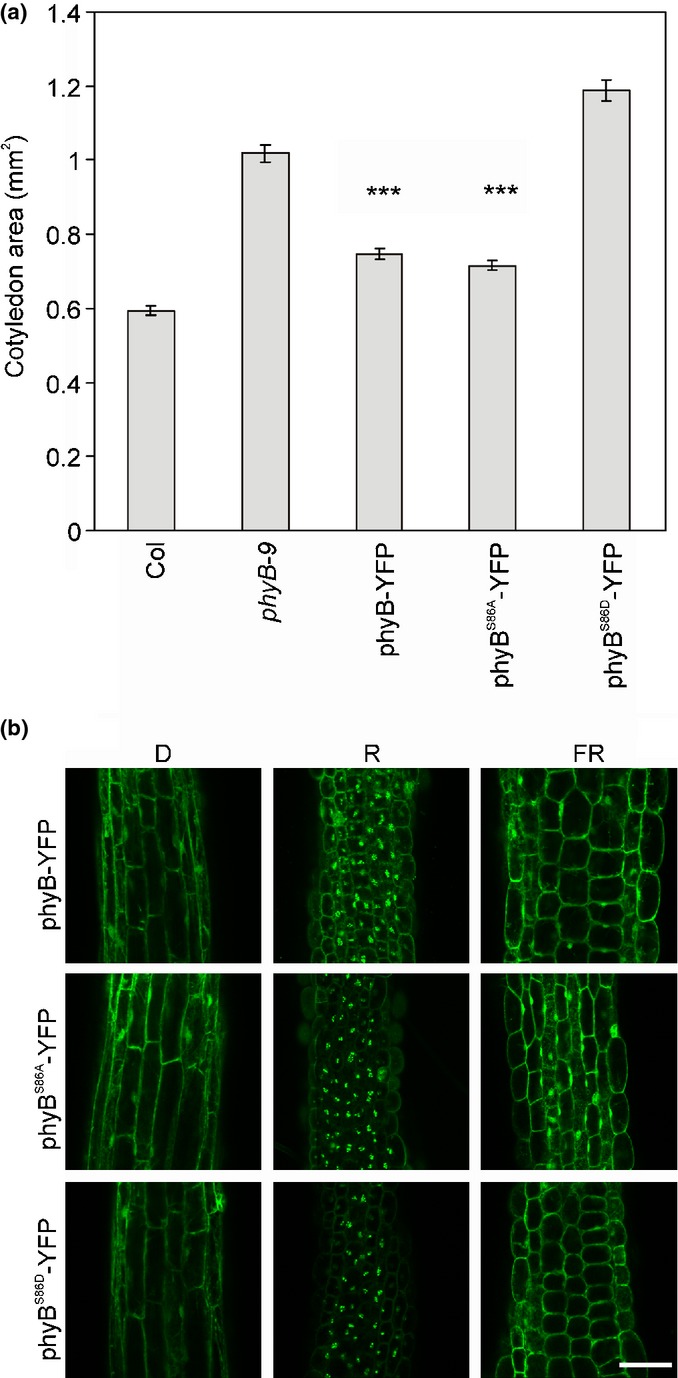
The phosphorylation state of *Arabidopsis thaliana* phyB S86 modifies nuclear localization and signaling when low Pfr : Ptot ratio is available. (a) Cotyledon area of wild-type Columbia (Col), *phyB-9* mutant (*phyB-9*) and transgenic seedlings expressing either *35S:PHYB-YFP* (phyB-YFP) or *35S*:*PHYB*^*S*^^*86A*^*-YFP* (phyB^S^^86A^-YFP) or *35S*:*PHYB*^*S*^^*86D*^*-YFP* (phyB^S^^86D^-YFP) transgenes in *phyB-9* background is shown. The seedlings were grown for 4 d under 10 μmol m^2^ s^−1^ FR light. *n *> 30; error bars, ± SE; ***, *P* < 0.001, compared with *phyB-9* mutant. (b) Intracellular localization of wild-type or mutant phyB-YFP chimera proteins. Confocal laser scanning microscopy images of hypocotyl cells were taken after 4 d of growth in darkness (D) or under 50 μmol m^2^ s^−1^ red light (R) or 10 μmol m^2^ s^−1^ far-red light (FR). Bar, 75 μm.

This novel hypothesis could result in a paradigm shift regarding our view about the molecular mechanisms mediating phyB nuclear import. For example, a very recent study reported that several NLS-possessing core components of the plant circadian clock bind directly to phyB in yeast-2-hybrid assays and transiently transformed protoplasts (Yeom *et al*., 2014). Interestingly, the CIRCADIAN CLOCK ASSOCIATED 1 and TIMING OF CAB EXPRESSION 1 bind to phyB Pr, LUX ARRHYTHMO binds to phyB Pfr, whereas LATE ELONGATED HYPOCOTYL 1, EARLY FLOWERING 3 and GIGANTEA bind to both phyB conformers. These interactions can potentially modulate accumulation thus the available phyB pool for signaling in the nucleus and could also explain why phyB is not fully excluded from the nucleus even in darkness. The hypothesis, namely that phyB nuclear import is mediated by a wide array of phyB-interacting proteins is also circumstantially supported by another recent report. Zheng *et al*. (2013) found that SPA1 protein binds to phyB Pfr and facilitates its nuclear import under continuous FR irradiation, when the Pfr : Ptot ratio is extremely low. As the FR-induced accumulation of phyB appears to be phyA–independent, these authors concluded that SPA1 captures the low number of phyB Pfr conformers produced by cFR illumination, which then downregulates phyA signaling under these conditions (Zheng *et al*., 2013). We show here that phosphorylation of phyB S86 can modify this response. Fig.2 demonstrates that nonphosphorylated phyB^S86A^ accumulates to higher levels in the nucleus and inhibits cotyledon expansion more efficiently in cFR when compared with phyB^S86D^ mutant mimicking constant phosphorylation. These observations are readily explained by the fact that phosphorylation of S86 accelerates dark reversion of phyB Pfr (Medzihradszky *et al*., 2013), that is, it further reduces the low number of phyB^S86D^ Pfr produced by cFR, available for signaling. It is important to note that import of phyB Pfr into the nucleus is inhibited by FR at high Pfr : Ptot ratio, thus our observation also supports the model by Zheng *et al*. (2013) which postulates that translocation of phyB into the nucleus is mediated by different molecular mechanisms under different light conditions. However, it is often the case that novel findings answer some questions but provoke many more new ones. This is also true for the new concept explaining light-dependent translocation of phyB to the nucleus. First, at present it would be premature to exclude the possibility that phyB does not contain NLS-like motif(s) as it is possible that the importin(s) interacting with phyB *in planta* is not conserved in the heterologous *Acetabularia* system. Second, it is fair to say that we do not understand how the action of the seemingly numerous phyB-interacting proteins is regulated and integrated to ensure optimal phyB signaling under different conditions.

## Molecular mechanism of PHYC-E translocation to the nucleus

The role of Arabidopsis phyC, phyD, phyE is less pronounced in the R/FR-driven photomorphogenesis when compared with phyB, but it was shown that these phytochromes also translocate into the nucleus (Kircher *et al*., 2002). However, more recently it was reported that phyB and phyD can heterodimerize with each other and phyC and phyE *in vitro* and that the vast majority of phyC, phyD and phyE exist and function as phyB-phyC, phyB-phyD, etc. heterodimers *in planta* (Clack *et al*., 2009). It follows that in the early studies, although phyC, phyD and phyE were overexpressed by using the strong viral *35S* promoter, researchers monitored not only the subcellular distribution of phyD, phyE homodimers but very likely also the subcellular distribution of phyB-phyC, phyB-phyD and phyB-phyE heterodimers. A more recent study investigated subcellular localization of phyC, phyD and phyE in various phytochrome mutants lacking phyB, phyD or both (Adam *et al*., 2013). It was found that in the absence of phyB and phyD, overexpressed phyE forms homodimers *in planta*, these homodimers are functional and regulate a subset of photomorphogenic responses, and phyE is transported into the nucleus at very low fluences of R light (Adam *et al*., 2013). This process is very effective and saturated at low fluences, similarly to phyA and contrary to phyB. However, unlike phyA, accumulation of phyE in the nucleus is independent of FHY1/FHL. phyE Pfr does not bind PIFs, thus we postulate that light-dependent import of phyE can also be mediated by a yet unknown molecular machinery distinctly different from those described above for phyA and phyB. We know much less about the molecular mechanism mediating nuclear import of phyC and phyD. R light slightly increases the nuclear pool of overexpressed phyC in *phyD* null mutants, but phyC functions only as phyB-phyC heterodimer, whereas nuclear accumulation of phyD is not regulated by R/FR light in the absence of phyB (Adam *et al*., 2013). Taken together, we conclude that our understanding of the mechanisms mediating subcellular distribution of homodimers of phyC, phyD and phyE or heterodimers of phyB-phyC, phyB-phyE, etc. is still in its infancy.

## Photobodies of phytochromes

phyA and phyB are not distributed equally in the nucleoplasm but associated with distinct sub-nuclear complexes termed photobodies (PBs). This is also true for phyC, phyD and phyE, but the number and size of phyC, phyD and phyE associated PBs is characteristically different when compared to phyB PBs (Adam *et al*., 2013). Recently, an excellent review covered the potential roles and composition of phytochrome PBs (Van Buskirk *et al*., 2012). Here we mainly focus on new insights and ongoing advancements about their functions.

## Dynamics and functions of early and transient photobodies

In dark-grown Arabidopsis seedlings phytochromes localize to the cytosol and, with the remarkably exception of phyA, to the nucleus. Kinetic analysis of cellular localisation showed that light leads to the formation of phyA-specific cytosolic SAP complexes (Kircher *et al*., 1999) which had been interpreted as sites of destruction of the light-labile receptor (Speth *et al*., 1987). Additionally, as described in the previous paragraphs, formation of phytochrome Pfr results in nuclear import of the photoreceptors and the appearance of at least two different types of PBs. Within minutes after the onset of light, numerous phyA- and phyB-containing complexes are formed which disappear rapidly during further irradiations. The mechanism underlying biogenesis of PBs is poorly understood. However, data published in a very recent study by using the nucleolus-tethering system (NoTS) suggest that assembly of photobodies may follow a self-organisation model (Liu *et al*., 2014). In the case of PHYB, formation of these early PBs is strictly dependent on PIF3 (Bauer *et al*., 2004). Detailed analysis demonstrated that (1) physical interaction of these molecules is mediated by specific binding sites (Khanna *et al*., 2004) which leads (2) to phosphorylation of the PIFs (Al-Sady *et al*., 2006) and subsequent degradation of these transcription factors. The tight correlation between the formation of phyB PBs and PIF3 degradation (Van Buskirk *et al*., 2012) and that the PB-deficient mutant *hmr*, is also defective in the degradation of PIF1 and PIF3 suggest that phyB PBs are required for PIF degradation (Galvao *et al*., 2012). However, the molecular mechanism by which phyB PBs mediate PIF degradation is still unclear.

## Dynamics of late type of photobodies correlate to physiological PHY functions

During extended irradiations a second, late type of nuclear bodies of PHY with a diameter up to one micrometer is formed (Kircher *et al*., 1999, 2002; Yamaguchi *et al*., 1999). Remarkably, the light requirements for the dynamic formation of these PBs and physiological functions of *PHYA* and *PHYB* seem to be strictly correlated (Kircher *et al*., 1999, 2002). The close relationship of light-dependent localization dynamics and physiological response has been extended by a very recent study. The authors demonstrate that the *PHYB*-mediated shade-avoidance syndrome (SAS) – which allows the plants to escape neighbors competing for photosynthetic active radiation – and the dynamics of PB pattern formation appear to be closely related. Transfer of plants to light environments comprising low R : FR light ratios as well as into low-light conditions lead to a significant and reversible increase of the amount of phyB photobodies (Trupkin *et al*., 2014). Interestingly, the nuclear localization pattern under such conditions exhibits changes not only in number but also in size distribution of phyB PBs, a phenomenon which had been described before by analysing hypocotyl growth regulation under varying fluence rates of light (Chen *et al*., 2003). In summary, these observations together with those reporting that mutated versions of phytochromes with aberrant physiological properties also exhibit abnormal localization patterns (e.g. (Kircher *et al*., 2002; Matsushita *et al*., 2003; Medzihradszky *et al*., 2013) led to the hypothesis that the late type of PBs are functionally relevant structures for phytochrome signaling pathways.

## Photobodies as storage pool of active PHYB

The exact molecular function of the later type of nuclear structures is still under debate. It has been proposed that late-type PBs of phyB could be involved in the slow process of light-dependent degradation of the photoreceptor (Sharrock & Clack, 2002) Although no clear evidence for this assumption had been provided so far, it is striking that the half-life of phyB destruction and NB formation are similar. Additionally, some important negative acting components of light signaling pathways do also localize to sub-nuclear structures, among these COP1 and SPA proteins. These factors form COP1-SPA E3 ubiquitin ligase complexes, degrade positive acting elements in light signaling in darkness, but are also discussed to be conditionally involved in phyA degradation (Debrieux *et al*., 2013). A study combining predictive modeling with experimental approaches including analysis of physiological, photochemical and localization dynamics of phyB showed that phyB-associated PBs can serve as nuclear storage sites for active phyB Pfr (Rausenberger *et al*., 2010). These authors calculated that at defined exchange rates between the nucleoplasm and PB localized phyB even a slightly reduced rate of dark reversion of phyB in the PBs can extend the activity of the photoreceptor after light-to-dark transitions. Comparative analysis of hypocotyl growth control, localization patterns of wild-type and mutant photoreceptors, as well as phyB-controlled accumulation of PIF3 and selected target genes of the TF validated this hypothesis (Van Buskirk *et al*., 2014). In other words these authors provided strong evidence that late-type PBs indeed confer sustained activity to phyB Pfr after light-to-dark transfer. Mechanistically, the underlying molecular processes may be similar as described for the early PBs and could be a common theme for growth control also under natural light–dark cycles (Soy *et al*., 2012).
